# A New Bibliometric Index Based on the Shape of the Citation Distribution

**DOI:** 10.1371/journal.pone.0115962

**Published:** 2014-12-26

**Authors:** Tommaso Lando, Lucio Bertoli-Barsotti

**Affiliations:** 1 Department of Finance, VŠB Technical University of Ostrava, Ostrava, Czech Republic; 2 Dipartimento di Scienze aziendali, economiche e metodi quantitativi, University of Bergamo, Bergamo, Italy; Max Planck Society, Germany

## Abstract

In order to improve the *h*-index in terms of its accuracy and sensitivity to the form of the citation distribution, we propose the new bibliometric index 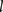. The basic idea is to define, for any author with a given number of citations, an “ideal” citation distribution which represents a benchmark in terms of number of papers and number of citations per publication, and to obtain an index which increases its value when the real citation distribution approaches its ideal form. The method is very general because the ideal distribution can be defined differently according to the main objective of the index. In this paper we propose to define it by a “squared-form” distribution: this is consistent with many popular bibliometric indices, which reach their maximum value when the distribution is basically a “square”. This approach generally rewards the more regular and reliable researchers, and it seems to be especially suitable for dealing with common situations such as applications for academic positions. To show the advantages of the 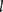-index some mathematical properties are proved and an application to real data is proposed.

## Introduction

The main success of the *h*-index [1] is probably due to its simplicity and its robustness, in that it is insensitive to low-impact publications with few or no citations. On the other hand, the drawbacks of the *h*-index have been discussed. Due to its symmetric structure [2], the *h*-index is insensitive to highly-cited publications: as soon as one such publication is part of the *h*-core (the group of the *h* most highly-cited papers; [3]), its actual number of citations no longer has an influence. Moreover, the number *h* alone seems to be too poor to discriminate among authors with similar scientific productions. This problem is known as the “low resolution” [4] of the Hirsch index: indeed, it is quite common to find researchers with equal *h* values. For these various reasons, several methods to complement or to improve the *h*-index have been proposed. The *A*-index [5], the *R*-index [6] and the *e*-index [4] complement the *h*-index by measuring the overall citation “intensity” in the *h*-core. On the other hand, the main stand-alone alternative to the *h-*index is probably the *g*-index [7], which is sensitive to exceptional publications, although it is not really sensitive to the form of the citation distribution. Other *h*-type indices attempt to improve the *h*-index by extracting additional information from the form of the citation distribution. We list some of these alternative approaches: the tapered *h*-index (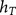, [8]); the Zynergy index (*z*-index) [9]; the recently introduced *h*′-index [10].

For a given author *x*, let 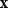 be his/her corresponding citation distribution - that is, the vector of non-negative integer components representing the number of citations per publication (as usual, in this paper we will assume that the citation distribution is sorted in decreasing order) - and let 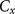 be the total number of citations. Our idea is to propose a new bibiometric index which depends on the *similarity* between the citation distribution 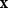 and a corresponding “ideal” distribution 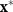, to be uniquely identified, under suitable constraints, in terms of i) number of papers; ii) number of citations per publication. More precisely, we search for an index which increases its value as the citation distribution 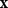 approaches the ideal form defined by 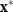. For instance, we could possibly define 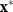 as a distribution with a “rectangular” form (henceforth we use this term to denote a vertical rectangle, i.e. most of the citations are “concentrated” on one or a few papers). This approach would reward researchers with a high impact on the scientific community (rather than regular productivity) and might be appropriate if it is necessary to evaluate high-level scientists (e.g. Nobel-prize winners or Fields medalists), but it could be misleading in many common contexts. In this paper we shall not follow this logic. In fact, study of the Hirsch index and its most important alternatives shows that the scientific performance of an author is always maximized if the distribution is basically represented by a “square” with side 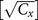 (where 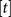 is the integer part of the number 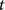): in this case we find that *h* and *g* (as shown in the next section) both reach their maximum values, as well as other bibliometric indicators. For this reason, in this paper we choose to define 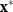 on the basis of a “squared” form. This idea yields a bibliometric index which is especially suitable for evaluating the scientific performance of “standard level” researchers. Consider the common case when the evaluation of a researcher is intended to assess his/her suitability for an academic position, e.g. as full professor etc. We believe that in such situations bibliometric indicators are especially useful. If applicants are similar/comparable, we believe that a bibliometric index should reward the more regular researchers in order to enable research institutions to make reliable selections. Thus, in a bibliometric context, a sort of “risk-averse” attitude suggests choosing, between researchers of the “same level” (that is, with equal or similar number of citations), the one who produces a good number of good quality papers, and who therefore has a more regular (i.e. “squared”) distribution of citations.

Although a general class of indices is proposed, we subsequently focus on a particular index, defined as 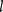. The mathematical properties of 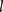 are presented and formally proved: 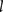 is a novel bibliometric indicator which outperforms the *h*-index in terms of accuracy and sensitivity to the form of the citation distribution. An application to real data shows that 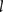 is strongly correlated with other important *h*-type indices. Moreover, we attempt to analyze the dependence between bibliometric rankings and the judgements of a committee, obtaining interesting results for the new index 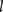.

## Methods

For a given researcher *x* with a total number of publications 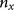 let us denote with 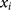 the number of citations of paper *i* (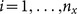), and let the papers be ranked in decreasing order according to the number of citations that they have received, so that 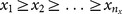. Let us denote the vector 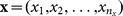 by the *citation distribution*. Henceforth let us call *a*-*core* (for any positive integer *a*) the set of the *a* most cited papers (if it exists). A bibliometric index of author *x* is a mathematical function of his/her citation distribution 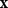.

The *h*-index [1] is defined as follows:
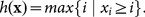
(1)


The number *h* identifies a set of significant papers, the so-called *h-core*. It is interesting to observe that the Hirsch index mainly depends on the form of the citation distribution: *h* is greater when the distribution is “squared” and smaller when the distribution has a “rectangular” form. In particular, 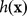 cannot exceed 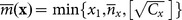 where 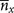 is the number of papers with at least 1 citation [11]. *A fortiori*, for any author 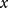 with a fixed number of total citations 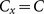 the value of 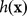 cannot exceed 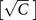. In particular, the distribution 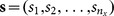, 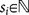, with total citations 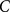 and such that 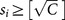 for 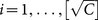 yields 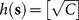. Note that 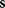 can be basically represented by a “square” with side 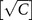. To be more specific, we can say that, for any possible citation distribution 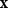 such that 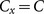:
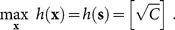
(2)


One of the main alternatives to the *h-*index is the *g*-index, proposed by Egghe [7]. The *g*-index is defined as:
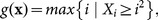
(3)where 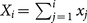. Similarly to *h*, the number *g* identifies a set of significant papers, the *g-core* (note that this set may be constituted by fictitious publications without citations, when 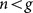; [12]). It is interesting to note that 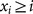 for 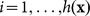 yields 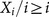 for 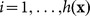; thus, by definition, the *h*-core is a subset of the *g-*core (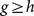, as is well known). The *g-*index is sensitive to highly-cited publications and does not strictly depend on the form of the distribution. Indeed it is known that *g* is sensitive to *concentrative transfers* [13], [12]. Hence, for a given number of total citations 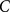, a distribution which concentrates all these citations on a single paper maximizes *g*. Actually, unlike *h*, the *g-*index can be maximized by both a “squared” and a “rectangular” distribution: from this point of view we can say that the *g*-index is more “flexible” than the *h-*index. On the other hand, this shows that *g* does not depend on the form of the distribution. This result can be proved as follows. Define by 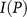 the logical function such that 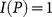 if the proposition 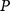 holds true and 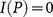 otherwise. For any author *x* with 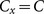 citations, consider the corresponding “rectangular” distribution: 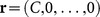 (vector with 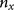 elements, for instance). Observe that:
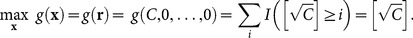
(4)


Let 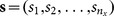 be the “squared form” distribution such that 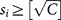 for 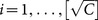 (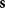 can be obtained from 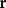 by a finite number of *elementary transfers*, called *T-transforms* in [14, p.32]. Consider that, for 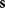, we obtain 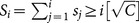; thus
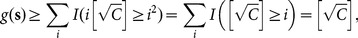
(5)hence 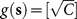. We conclude that 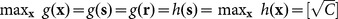. Note that this results can also be derived from the bounds of the *h*- and *g*-indices recently studied by [15].

Overall, it seems that both indices (*h* and *g*) agree when the citation distribution is squared, which happens when a researcher produces a significant number of good quality publications, rather than a few outstanding ones. As a consequence of this idea, which is apparently consistent with the most popular bibliometric indices, we propose to measure the scientific performance of a researcher by comparing his/her citation distribution to a squared benchmark distribution, as described in the next subsection.

### Defining an “ideal” citation distribution

Define 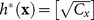. The number 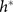 corresponds to a set of papers which includes the *h-*core as well as the *g*-core. It is worth noting that it may happen that an author does not have 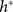 published papers (i.e. when 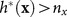 which is quite uncommon, especially for “standard” researchers): we may consider 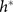 as an “ideal” number of papers. If author 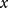 with 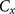 citations has at least 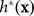 publications, then (according to the citation distribution) he/she can maximize his/her scientific performance (in terms of both *h* and *g*); otherwise he/she cannot. In the literature, several methods have been proposed to select the optimal number of significant or “elite” papers which have a high impact on the scientific community. Generally, bibliometric indicators based on larger sets are more appropriate to measure the overall performance instead of scientific impact. On the other hand, indices that focus on a smaller set or “core” of highly cited papers assess authors based on their impact, overlooking the regularity of their performance. The 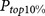 index [16], [17] is the number of papers which belong among the top 10% highly cited publications on the same subject and in the same year; obviously by varying the percentage we can obtain more or less restricted elite sets. One of the main advantages of this approach is that it makes it possible to compare authors in different research fields and different periods of time. Nevertheless, the aim of the 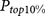 index is quite different from ours, and we do not have available the data for its computation; for these reasons the 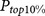 is not included in our analysis. The *π*-index [18], [19] is obtained from the citations within the *π*-core, that is, the set of the most 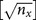 cited papers. Generally, the *π*-index considers the most elite papers and therefore rewards papers of high impact, although the *π*-core depends on the number of publications, which is not a measure of impact itself. Moreover, other indicators such as the above mentioned *A*-, *R*- and *e*-indices are based on the number of citations within a generally larger set i.e. the *h*-core. Note that these indices have been proposed as complementary to 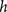 and not as “stand-alone” indicators due to some possible drawbacks (e.g. an increase in 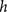 could produce a decrease in 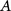 or 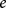). The aim of this paper is to take into account not only the impact but also the regularity of an author during his/her entire career. In fact, as mentioned above and confirmed by our case study, we are interested in assessing “standard level” researchers who possibly do not have outstandingly higlhy cited papers. We therefore propose to consider the *h* ^*^-core, which generally includes the *h*-core, as well as the *π*-core.

As discussed above, the *h*- and *g*-indices can be maximized by a “squared” citation distribution (with side equal to 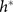). It is worth noting that, for a fixed number of citations 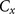, a distribution of this kind also maximizes other alternative *h*-type indices, such as the 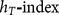 [8] and the *R*-index [6]. Therefore, some of the most important bibliometric indices suggest that a “squared-form” citation distribution should represent an “ideal” for an author. Also, the *z*-index [9] complies with this principle, because 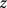 increases with consistency (regularity, see [20]). We have maximum consistency in the case of absolutely uniform performance [24], that is, when all the papers have an equal number of citations. We believe that the best performance can be achieved when a combination of impact (citations per paper), productivity (number of papers) and consistency is maximized, and this happens with a “squared” distribution. In particular, we propose to define an ideal number of citations per paper as described below.

Assume that author 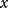 has at least one publication and one citation. Define 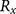 as the natural number such that 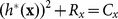. Given 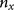, 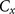 and 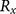 we can now define an ideal citation distribution, say 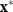, such that 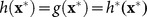. Although there may be different (also easier) ways to define 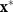, we propose choosing the distribution 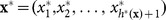 (a vector with 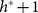 components) which reflects maximal regularity, in that 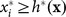 as long as possible (for 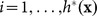) and 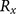 is symmetrically equidistributed among papers/citations. This idea is formalized as follows:
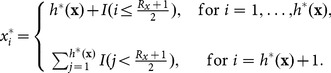
(6)


Thus, the components of 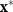 are all positive integer numbers except for the last one (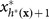), which can possibly be 0. The choice of a vector 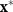 with 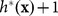 components instead of 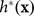 is due to the fact that, with this choice, we can “distribute” 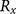 in the most efficient way in order to maximize the most important bibliometric indices. Let the symbol “
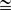
” represent a generalized equality between vectors which simply excludes the zero-elements from 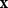 (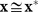 if 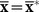, where for a *k*-dimensional citation vector 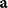, define 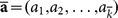 and 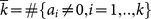). 
Note that the citation distribution defined by 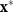 maximizes *h*, *g* and also the 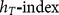 [8], so that it is evident that any researcher *x* for whom 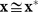 really optimizes his/her scientific performance.

### A bibliometric index based on the form of the citation distribution

For any author *x*, it is now possible to obtain a class of bibliometric indices which are sensitive to the similarity between the real distribution 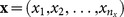 and the corresponding ideal distribution 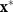. The basic idea is that, between two scientists *x* and *y* of the same level, i.e. with the same number of total citations 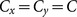, the one (say *x*) whose distribution 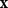 is more “similar” to 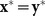 should be preferred (it is easier for author *x* to reach his/her maximum *h-* and *g-* values 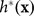 compared to *y*).

Denote by 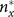 the number of papers such that 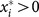 (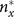 can be equal to 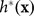 or to 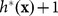 depending on 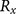) and assume that, in the rare case when 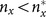, 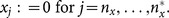 Drawing inspiration from statistical divergence measures between distributions [21], we can measure the “distance” between 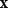 and 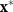 by analyzing the ratios 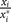, for 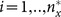: if they are (on average) close to 1, we can conclude that 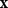 is close to 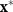. Suppose that the citation distributions 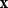 and 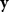 yield the same ideal distribution 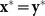. In order to determine whether 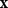 or 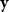 is closer to 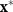 we can compare the ratio-vectors 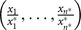 and 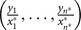 (where 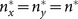): in particular, we should choose the distribution corresponding to the ratio-vector whose components are more “equal” or less “spread out”. From majorization theory [14] we can identify the class of functions which are consistent with this principle by a weighted sum of increasing and concave functions of the ratios 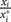.

In particular, we propose:
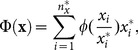
(7)where 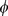 is increasing, concave but also positive and defined in 0. In the trivial case where a researcher has not received any citation (or published any paper), assume 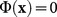.

It is of interest to note the relation between any function 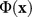 and the *relative majorization* (*r-majorization*) pre-order defined by Joe [22]. Suppose that 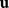 and 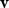 yield the same ideal distribution, say 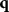 (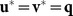), and let 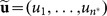, 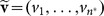 (where 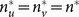) so that 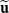, 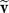 and 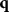 have an equal number of elements 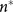. Moreover, suppose that 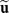, 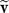 satisfy 
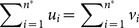
 (equal citations within the 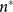-core). In such a matching situation, the relation 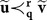, literally “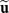 is *r-majorized* by 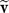 with respect to 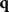
”, means that 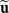 is closer to 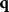 than 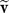: thus 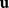 should be preferred to 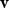 (according to the basic logic set out in the previous subsection). It is proved that 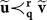 if and only if 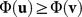 (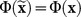 for any 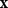) for any concave function 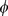 (note that this corresponds to the usual definition of *r-*majorization if we take 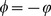, where 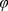 is convex). 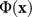 is said to be “order-preserving”, “isotonic” [14, p.19] or *Schur-concave* with *r*-majorization [22], which means that if 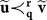 holds, then 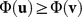. In particular, 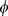 is also non-decreasing because we cannot allow 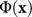 to decrease if an element of 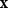 increases (i.e. additional citations).


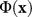
 is based on the ratio between real/ideal citations per paper within the ideal set of citations i.e. the 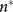-core. It is interesting to note the uncommon case when an author does not have enough publications i.e. 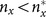, which simply yields 
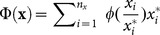
 (the number of addends is inferior since we assumed that 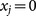 for 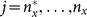). Thus 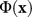 is indeed sensitive to the number of published papers. Moreover, the risk of considering papers which are not significant is countered by the fact that, if a paper has a low number of citations, the weight of those citations in 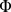 is downsized. On the other hand, 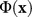 is also sensitive to highly-cited papers, because 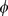 is increasing. Nevertheless, for a fixed value of 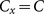, we obtain the best performance when 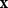 approaches 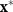, thus when the form of the distribution is “squared”: this is consistent with respect to the basic logic of many bibliometric indices including the *h*-index (especially) and also the *g*-index (as proved above).

Within the general class defined by 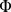, we choose 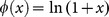 (increasing, concave, positive and defined in 0), which yields:
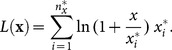
(8)


Finally, note that 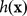 and 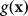 are integer numbers defined on the interval 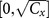. Thus, in order to obtain a bibliometric index which takes values within the same interval as the most popular ones (*h* and *g*), which can be useful for comparisons, we propose to normalize 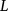 as follows:
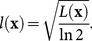
(9)


Note that 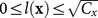, while *h* and *g* actually take values in 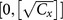.


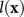
 is based on a sum of a particular function that we denote by 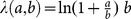 (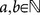 and 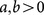). In the S1 Appendix, we prove (*Lemma 1*) that 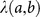 is an increasing function of *b* (as well as *a*, obviously). This justifies and motivates the choice of 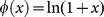. The *l*-index outperforms the *h*-index in terms of precision and accuracy with respect to additional citations and sensitivity to the shape of the distribution. Moreover, *l* is (like *h*) robust with respect to citations in the set of non-significant papers. In particular, in the S1 Appendix the following properties are proved.

**Property 1. Strict monotonicity with respect to citations**


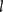
 is an increasing function of any additional citation.

**Property 2. Robustness with respect to non-relevant citations**

An additional citation within the *n* ^*^-core is always “heavier” than an additional citation outside the *n* ^*^-core.

**Property 3. Sensitivity to regularity**

An additional citation within the *h* ^*^-core is “heavier”, the closer the cited paper is to the *h* ^*^-th paper.

**Property 4. Sensitivity to elementary transfers**

If 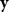 can be obtained from 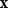 by an elementary transfer of citations between two papers in the *h* ^*^-core, then 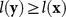.

## Results

The main purpose of the paper is to find an index which improves the *h*-index in terms of its accuracy and sensitivity to both: i) citation “intensity” in the set of most significant papers; ii) the form of the citation distribution. For this reason, it is interesting to study the relations between 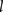 and some of the main alternatives to the Hirsch index (including the *g*-index and the 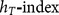).

### Theoretical examples

To verify the behavior of 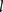 we re-propose the theoretical examples provided by Vinkler [23], which illustrate the advantages and disadvantages of the *h*-index. The same particular cases were used by [8] to show the accuracy of the 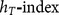. Before starting to analyze the results, we would point out that most of these theoretical datasets present quite uncommon features because they satisfy 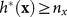. For this reason, in the next subsection we propose an application to real data.

The results in Table 1 show that the 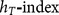 improves the *h*-index (as already argued in [8]) by measuring both the quality and quantity of publications, but it is not very sensitive to highly-cited papers. For this reason, we also compute indices which are mainly aimed at assessing scientific impact such as the *g*-index, the *p*-index [24], the *π*-index and the *R*-index. Note that 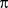 and 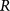 are both based on the number of citations within a set of elite papers (respectively the *π*-core and the *h*-core). Moreover, we consider the *z*-index, an impact measure which is also sensitive to the form of the citation distribution and rewards regular (consistent) scientific performances.

**Table 1:** Theoretical examples.

pap.\ aut.	A	B	C	D	E	F
1	100	9	10	50	9	10
2	98	8	10	50	8	110
3	98	8	10	50	7	100
4	97	6	10	50	6	90
5	96	5	10	50	5	80
6	4	4	10	50	_	_
7	3	4	10	50	_	_
8	2	3	10	50	_	_
9	1	2	10	50	_	_
10	1	1	10	50	_	_
n	10	10	10	10	5	5
C	500	50	100	500	35	500
h	5	5	10	10	5	5
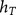	13.27	6.89	10	18.5	5.79	12.46
g	22	6	10	22	5	22
R	22.11	6	10	22.36	5.91	22.36
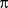	2.96	0.25	0.3	1.5	0.17	2.3
p	29.24	6.29	10	29.24	6.25	36.84
z	23.54	5.82	10	29.24	6.17	36.59
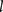	17.03	6.7	10	19.54	5.76	16.64

Authors  = A, B, F; *n* =  number of papers; *C* =  tot. number of citations.

On analyzing Table 1, first to be noted is that the *g*- and *R*-indices yield very similar results. More importantly, consider authors D and F: the 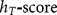 of author D is significantly higher than the 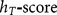 of author F. Conversely, the *g*-index is sensitive to the most cited papers but ignores the form of the citation distribution (authors A, D and F are equivalent according to their *g-*scores). Table 1 also shows that the *π*-index reflects scientific impact more accurately compared with the *g*-index in that it ranks author A above all the others and author F above author D. Indeed, on taking into consideration only the citations of the elite papers (i.e. the *π*-core), the *π*-index rewards a few papers of high impact in spite of poor regularity or consistency. Also note that, as mentioned above, 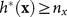, so that every paper of every author (from A to F) belongs to the *h* ^*^-core; on the other hand, the number of elite papers considered for the computation of 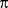 is significantly smaller (e.g. 3 vs. 10 for author A), for this reason in this particular case the difference between 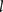 and 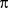 is especially accentuated.

The *z*-index behaves similarly to 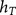 and 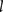 if 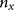 is equal, this is because 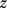 is sensitive to the form (regularity). Conversely, when authors have similar numbers of citations but different numbers of published papers, a smaller number of papers may enhance the performance. In fact, the formula of the *z*-index is based on the product between a consistency measure and an impact measure, which is the *p*-index. In turn, the *p*-index is based on the ratio 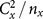, where the number of papers is the denominator. Hence, among the considered indices, only *p* and *z* rank author F above the others, this is not just because of his/her number of citations but also because his/her number of papers is half that of the others.

The *l*-index seems to be “halfway” between the 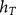 -index and other impact measures because it is sensitive to both the form of the distribution and the number of citations of the most cited papers. Indeed consider again authors D and F: according to 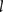 the gap between the scores of author D and author F is considerably reduced. On the other hand, 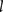 and 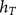 provide similar results when authors do not have highly-cited papers (authors B, E). The proposed *l*-index is strictly related to the 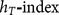: 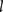 is sensitive to the “closeness” to the ideal distribution 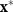 which, as mentioned in the previous section, maximizes the 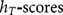. Nevertheless, there are some significant differences between 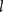 and 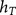. Besides being sensitive to a “squared” form of the citation distribution, 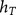 is also *symmetric* (property defined by Kongo, [2]) while 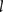 does not fulfill the symmetry property (for Property 2 defined in the previous section). Indeed, to avoid any misunderstanding, we now prove that 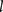 and 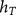 are not *monotonically related* [25] with a straightforward counter-example. Consider 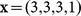 and 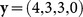: in this case 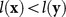 but 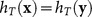. The *l*-index could be an improvement of 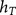 because it is sensitive to any additional citation and downsizes the effect of highly-cited papers (like 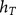); on the other hand, it is not “symmetric” because the weight of the papers outside the 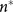 core (non-significant) is lower than the weight of the most cited ones (significant).

### Case study

The Italian National Scientific Qualification (Abilitazione Scientifica Nazionale, ASN) is a new procedure, based on scientific qualification criteria, for the recruitment of academic staff in Italy. The ASN has involved tens of thousands of candidates (approximately 40,000). Here we focus on the set of 149 physicists who were applicants in the 2012 ASN for a full professorship in the specific area of Condensed Matter Physics. An expert panel of evaluators (a Committee of five members) was asked, by the Italian University Ministry, to approve (“habilitate”) or to reject each candidate. In Italy, habilitation is necessary to be eligible for a full professorship. The goal of the Committee was to select the best candidates by taking the impact of their scientific research into account.

The complete list of publications and corresponding citations for each of these applicants was retrieved by us from Scopus in January 2014. From the original (autoselected) sample of 149 datasets (for almost all the candidates for full professorship the status was that of “Associate Professor”; the list of candidates was retrieved from the URL: http://abilitazione.miur.it/public/index.php), 18 datasets were discarded from the analyses due to insufficient citation data (e.g. an *h*-index less than 2) or difficulties in identifying the scientist. Then, for each of the 131 selected datasets, several different research productivity indices were computed, including 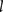. We analyzed the results of *h*, *g*, 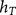, 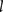, but also 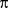, 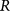, 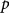, 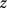 and the *h*′-index, recently proposed by Zhang as an index “based on the citation distribution” [10]. Moreover, we computed some simple bibliometric indicators such as the number of the citations of the most cited (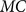) paper, the total number of citations 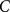, the total number of papers 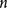 and the average number of citations per paper 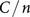. In Table 2 we present some descriptive statistics of the data. First to be noted is that, among 131 scientists, only 4 have a citation distribution such that 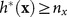, confirming that this is a quite uncommon situation. However, for all the authors the total number of papers is always smaller than the number of citations, and also 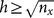 except for only 2 of them. We therefore argue that, generally, the *h* ^*^-core includes the *h*-core, which in turn includes the *π*-core. Hence, in this situation the *π*-index is focused on the most elite papers (and therefore focused on impact), while the *R*-index, and consequently the *l*-index, considers larger sets of significant papers.

**Table 2:** Descriptive statistics.

pap.\ aut.	min	max	Mean	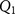	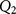	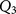	SK	SD	CV
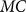	5	3068	358	104.5	177	328	3.16	542	1.51
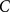	18	13916	2206	1156	1786	2716	2.49	1934.8	0.87
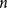	7	405	102	66	92	123	1.68	62.9	0.62
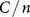	1.53	83.5	21.18	12.68	17.9	25.82	1.88	14.36	0.67
h	2	53	21.63	18	22	27	−0.10	8.66	0.40
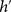	1.5	108.7	32.5	19.82	29.6	43	1.19	19.39	0.59
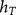	4.07	92.87	36.28	30.51	36.76	45	0.10	14.67	0.40
g	3	100	39.71	29	40	48.75	0.53	18.16	0.46
R	3.31	102.07	37.02	26.14	36.72	44.06	0.71	17.3	0.47
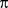	0.08	74.26	11.77	4.81	8.37	13.8	0.98	12	1.02
p	3.59	90.18	33.36	23.8	32.1	39.14	0.91	16.1	0.48
z	3.02	39.43	19.76	16	20.2	24.8	−0.14	7.36	0.3
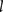	4.03	98.73	37.51	30	37.82	46.3	0.26	15.56	0.41

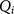
  = i-th quartile (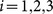), 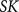  =  Skewness, 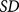  =  Standard Deviation, 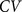  =  Coefficient of Variation, 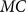  =  Maximum number of citations (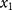).

We also compared the results in terms of correlations between indices. Since in our opinion all those indices should be considered as measures at the level of *ordinal scale* and not *interval scale* (the critical question here is if the “difference” between, for example, two consecutive values of the *h*-index, 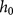 and 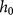 +1 scale, expresses the same “gap” regardless of the value of the baseline level 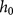), these data should be analyzed only by using nonparametric methods for ordinal data. In particular, Table 3 presents the Spearman correlation coefficient (that is, the Pearson correlation coefficient between the ranked variables) for each pair of indices considered. As can be seen, the 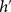-index yields results which are not quite consistent with those of the other indices, in particular its correlation with the productivity index (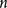) is really low. More importantly, some indices show good correlation with 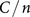 and therefore can be considered as impact measures: this set of indices consists of 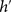, 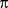, 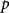, 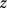, 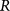 and 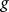 (interestingly, 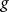 and 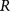 present very similar results, as already argued in [20]). In particular, some of these indices (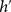, 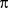, 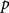, 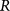 and 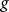) are also highly correlated with 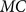, then, we argue that their values could be distorted by a single highly cited paper. On the other hand, 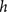 and 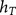 are also sensitive to the productivity, since they show good correlation with 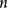. The *l*-index is highly correlated with both types of indices. Therefore, as hypothesized in the previous subsection, our data confirm that 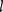 is a good compromise for measuring both impact and form, indeed, it is especially appropriate for assessing authors based on the impact of their most cited papers as well as the regularity of their scientific production. To strengthen our thesis, it is also interesting to note that 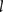 is the index most correlated with 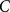 (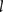 is a strictly increasing function of any additional citation, see property 1) and the second most highly correlated with 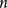 (after 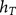).

**Table 3:** Spearman correlation coefficients.

	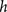	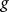	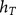	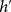	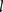	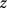	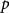	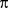	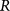	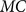	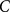	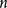	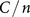
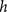	1.000												
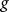	0.897	1.000											
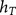	0.971	0.920	1.000										
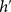	0.674	0.858	0.669	1.000									
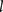	0.964	0.968	0.980	0.754	1.000								
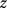	0.871	0.863	0.853	0.769	0.872	1.000							
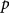	0.851	0.978	0.868	0.903	0.926	0.902	1.000						
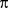	0.834	0.982	0.876	0.860	0.931	0.786	0.955	1.000					
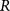	0.885	0.998	0.907	0.877	0.958	0.860	0.982	0.984	1.000				
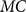	0.628	0.847	0.670	0.877	0.752	0.634	0.866	0.896	0.860	1.000			
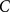	0.927	0.985	0.955	0.779	0.986	0.850	0.947	0.966	0.979	0.802	1.000		
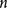	0.764	0.621	0.790	0.259	0.737	0.479	0.499	0.603	0.597	0.351	0.710	1.000	
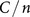	0.624	0.803	0.622	0.944	0.702	0.819	0.892	0.786	0.820	0.834	0.728	0.153	1.000

Spearman correlation coefficients between bibliometric indicators.

Let us define the dichotomous “habilitation” variable, with values 0 ( =  rejected applicant) and 1 ( =  approved applicant). It is interesting to study the dependence between these indices and the judgements of the Committee (note that 69% of the 131 applicants were approved by the Committee). Table 4 reports the values of the Spearman correlation between the five indices considered and the habilitiation variable. Indices 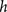, 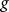, 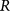 and 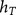 show similar and good results in terms of coherence with the judgements; similar but slightly less satisfactory results are obtained for 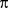, 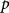 and 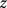; while the *h*′-index seem to be less associated with the habilitiation variable. Moreover, 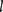 is slightly more correlated with the habilitation variable than are *h*, *g*, 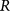 and 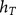. Hence, we may suppose that 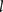, which rewards reliability as well as the impact on the scientific community, reflects the evaluation criteria of the Committee in a quite satisfactory manner. Moreover, after subdividing the sample into “approved” and “rejected” applicants, the *W* statistic for the two-sample Wilcoxon rank sum test [26] was also computed for each of the indices considered. We recall that the purpose of this test is to compare the ranks of one of the sub-samples (we considered that of the “approved” applicants: 91 cases) with those that would be expected if the null hypothesis of equal distribution of the levels of the index considered were true. The alternative is a condition of stochastic dominance, and, in our case, the null hypothesis was rejected for large values of *W*. Hence, one would expect higher values of *W* for the indices more in agreement with the Committee's judgement. Interestingly, as can be seen in Table 4, the Wilcoxon statistic *W* is strictly coherent with all the above results.

**Table 4:** Analysis of the habilitation variable.

	*h*′	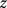	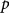	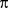	*h*	*g*	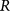	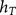	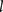
*HAB*	0.450	0.533	0.569	0.576	0.590	0.S592	0.593	0.594	0.611
*W*	7033.0	7223	7304	7320	7349.0	7355.5	7361	7360.5	7400.0

First row: Spearman rank order correlation coefficients between the variable *HAB*" and various bibliometric indicators. Second row: Wilcoxon rank sum statistic (with reference to the cases in the larger of the two samples).

## Conclusion

We have proposed a general method for improving the *h*-index that is based on the form of the citation distribution. The approach consists in defining an ideal optimal citation distribution for any author: a good bibliometric index should be sensitive to the closeness of the real citation distribution to its ideal one. In particular, the 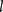-index is obtained when the reference distribution is “squared”. Theoretical properties and empirical results from real data have been studied thoroughly. 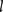 rewards reliability and regularity, but it is also sensitive to highly-cited papers: its use is especially appropriate to evaluating (for instance) applicants for university positions, which is a major issue within the field of scientometrics. In particular, the statistical analyses on our case study yielded some interesting results: bibliometric rankings were compared with the judgments of a committee and it seems that 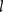 is the most appropriate (among the indices considered) for interpretation of this relation. Although the computation of 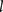 is not so simple (compared to the Hirsch index and some other popular bibliometric measures) the results of the paper are encouraging. They suggest that the new index could truly represent a significant alternative to the many existing *h*-type indices.

## Supporting Information

S1 Appendix **Proofs.**
(PDF)
