# Supplementary material for: A New Bibliometric Index Based on the Shape of the Citation Distribution
Source: PLoS One. 2014 Dec 26;9(12):e115962. doi: 10.1371/journal.pone.0115962 (PMC4277364; doi:10.1371/journal.pone.0115962)
Supplement: S1 Appendix — Proofs. (PDF) [file pone.0115962.s001.pdf]

Appendix S1. Proofs.

Lemma 1

If $a, b \in \mathbb{N}$ *and* $a, b > 0$*, then* $\lambda(a,b) = \ln(1+\frac{a}{b})\ b$ *is an increasing function of* b*.*

Proof

Since by hypothesis $\frac{a+b}{b} > 1$, and since $\partial_b(\lambda(a,b)) = \ln\left(\frac{a+b}{b}\right) + \frac{b}{a+b} - 1$, it is sufficient to prove that the function $\xi(z) = \ln(z) + z^{-1} - 1 > 0\ \forall z > 1$. This follows from the fact that $\xi(1) = 0$ and $\xi'(z) = (z-1) \cdot z^{-2} > 0\ \forall z > 1$.

Note: in the following proofs we refer without loss of generality to L instead of l: indeed, because l is a monotone transformation of L, the results hold true for both of them.

Denote with $\mathbf{u}_{\downarrow}$ the non-increasing re-arrangement of the vector $\mathbf{u}= (u_1, \ldots, u_n)$, so that if $\mathbf{v}= (v_1, \ldots, v_n) = \mathbf{u}_{\downarrow}$ then $v_1 \geq v_2 \geq \ldots \geq v_n$.

Property 1

If $\mathbf{x} = (x_1, \ldots, x_{n_x})$ *and* $\mathbf{y} = (x_1, .., x_k + 1, \ldots, x_{n_x})_{\downarrow}$ *then* $l(\mathbf{y}) > l(\mathbf{x})$*.*

Proof

Consider the two possible cases: 1) $k > n_x^*$ and 2) $k \leq n_x^*$.

Suppose $h^*(\mathbf{x}) = h^*(\mathbf{y}) = h^*$.

1. $L(\mathbf{y}) - L(\mathbf{x}) = \sum_{i=1}^{h^*+1} [\lambda(x_i, y_i^*) - \lambda(x_i, x_i^*)]$.

   As $C_y > C_x$ then $y_i^* \geq x_i^*$ for $i = 1, \ldots,\ h^*$ and there exists at least one element, say y_j^*, $j \in \{1, \ldots,\ h^*\}$, such that $y_j^* \geq x_j^*$. Thus Lemma 1 yields $\lambda(x_i, y_i^*) - \lambda(x_i, x_i^*) \geq 0$, for any $i = 1, \ldots,\ h^*$ and $\lambda\left(x_j, y_j^*\right) - \lambda\left(x_j, x_j^*\right) > 0$. Hence $L(\mathbf{y}) - L(\mathbf{x}) > 0$ and we obtain the thesis.

2. $L(\mathbf{y}) - L(\mathbf{x}) = \sum_{i \neq k} [\lambda(x_i, y_i^*) - \lambda(x_i, x_i^*)] + \lambda(x_k + 1, y_k^*) - \lambda(x_k, x_k^*)$, where $\sum_{i \neq k} [\lambda(x_i, y_i^*) - \lambda(x_i, x_i^*)] > 0$ for point i). Moreover, $\lambda(a,b)$ is also an increasing function of a, thus $\lambda(x_k + 1, y_k^*) - \lambda(x_k, x_k^*) > 0$ which yields the thesis.

Suppose $h^*(\mathbf{x}) < h^*(\mathbf{y})$, then the thesis holds true *a fortiori*.

Property 2

Let $\mathbf{x} = (x_1, \ldots, x_{n_x})$, $\mathbf{y} = (x_1, .., x_u + 1, \ldots, x_{n_x})_\downarrow$ *and* $\mathbf{w} = (x_1, .., x_v + 1, \ldots, x_n)_\downarrow$. *If* $u \leq n_x^* < v$, *then* $l(\mathbf{y}) > l(\mathbf{w})$.

Proof

Clearly, $\mathbf{y}^* = \mathbf{w}^*$ and $h^*(\mathbf{y}) = h^*(\mathbf{w}) = h^*$.

$$
\begin{aligned}
& L(\mathbf{y}) - L(\mathbf{w}) = \\
& = \sum_{i \neq u} \lambda(x_i, y_i^*) + \lambda(x_u + 1, y_u^*) - \sum_{i=1}^{h^*+1} \lambda(x_i, y_i^*) = \\
& = \sum_{i \neq u} \lambda(x_i, y_i^*) + \lambda(x_u + 1, y_u^*) - \sum_{i \neq u} \lambda(x_i, y_i^*) - \lambda(x_u, y_u^*) = \\
& = \lambda(x_u + 1, y_u^*) - \lambda(x_u, y_u^*) . \qquad (1)
\end{aligned}
$$

As $\lambda(a, b)$ is an increasing function of a, $\lambda(x_u + 1, y_u^*) - \lambda(x_u, y_u^*) > 0$ which yields the thesis.

Property 3

Let $\mathbf{x} = (x_1, \ldots, x_{n_x})$, $\mathbf{y} = (x_1, .., x_u + 1, \ldots, x_{n_x})_\downarrow$ *and* $\mathbf{w} = (x_1, .., x_v + 1, \ldots, x_{n_x})_\downarrow$ *and suppose* $x_v \neq x_u$. *If* $u < v \leq h^*(\mathbf{x})$ *then* $l(\mathbf{y}) < l(\mathbf{w})$.

Proof

Clearly, $\mathbf{y}^* = \mathbf{w}^*$ and $h^*(\mathbf{y}) = h^*(\mathbf{w}) = h^*$.

$$
\begin{aligned}
& L(\mathbf{y}) - L(\mathbf{w}) = \\
& = \sum_{i \neq k,l} \lambda(x_i, x_i^*) + \lambda(x_u + 1, x_u^*) + \lambda(x_v, x_v^*) + \\
& - \sum_{i \neq u,v} \lambda(x_i, x_i^*) - \lambda(x_u, x_u^*) - \lambda(x_v + 1, x_v^*) = \\
& = \lambda(x_u + 1, x_u^*) + \lambda(x_v, x_v^*) - \lambda(x_u, x_u^*) - \lambda(x_v + 1, x_v^*) = \\
& = \delta(u) - \delta(v) , \qquad (2)
\end{aligned}
$$

where $\delta(u) = \lambda(x_u + 1, x_u^*) - \lambda(x_u, x_u^*) = x_u^* \ln \frac{x_u^* + x_u + 1}{x_u^* + x_u}$ and $\delta(l) = \lambda(x_v + 1, x_v^*) - \lambda(x_v, x_v^*) = x_v^* \ln \frac{x_v^* + x_v + 1}{x_v^* + x_v}$. We need to prove that $\delta(u) - \delta(l) < 0$. Note that, for all integer numbers $m < n$, $\frac{m+1}{m} > \frac{n+1}{n}$. Thus, as $x_u^* \geq x_v^*$ and $x_u > x_v$ we derive that:

$$\frac{x_u^* + x_u + 1}{x_u^* + x_u} < \frac{x_v^* + x_v + 1}{x_v^* + x_v}, \tag{3}$$

which yields $\delta(u) < \delta(v)$ or equivalently $\delta(u) - \delta(v) < 0$.

Property 4

If $\mathbf{x} = (x_1, \ldots, x_{n_x})$ *and* $\mathbf{y} = (x_1, .., x_u - 1, \ldots, x_v + 1, \ldots, x_{n_x})_\downarrow$ *where* $u < v \leq h^*(\mathbf{x})$ *and* $x_v < x_u$*, then* $l(\mathbf{y}) \geq l(\mathbf{x})$*. Strict inequality holds if* $x_u - x_v \geq 2$.

Proof

The proof is similar to the proof of property 3.

Clearly, $\mathbf{y}^* = \mathbf{x}^*$ and $h^*(\mathbf{y}) = h^*(\mathbf{x}) = h^*$.

$$\begin{aligned}
&L(\mathbf{y}) - L(\mathbf{x}) = \\
&= \sum_{i \neq u,v} \lambda(x_i, x_i^*) + \lambda(x_u - 1, x_u^*) + \lambda(x_v + 1, x_v^*) + \\
&- \sum_{i \neq u,v} \lambda(x_i, x_i^*) - \lambda(x_u, x_u^*) - \lambda(x_v, x_v^*) = \\
&= \lambda(x_u - 1, x_u^*) - \lambda(x_u, x_u^*) + \lambda(x_v + 1, x_v^*) - \lambda(x_v, x_v^*) = \\
&= -\delta(u) + \delta(v),
\end{aligned} \tag{4}$$

where $\delta(k) = \lambda(x_u, x_u^*) - \lambda(x_u - 1, x_u^*) = x_u^* \ln \frac{(x_u^* + x_u - 1) + 1}{x_u^* + x_u - 1}$ and $\delta(l) = \lambda(x_v + 1, x_v^*) - \lambda(x_v, x_v^*) = x_v^* \ln \frac{x_v^* + x_v + 1}{x_v^* + x_v}$. We need to prove that $-\delta(u) + \delta(v) > 0$. For all integer numbers $m \leq n$, $\frac{m+1}{m} \geq \frac{n+1}{n}$. Thus, as $x_u^* \geq x_v^*$ and $x_u - 1 \geq x_v$ we derive that:

$$\frac{(x_u^* + x_u - 1) + 1}{x_u^* + x_u - 1} \leq \frac{x_v^* + x_v + 1}{x_v^* + x_v}, \tag{5}$$

which yields $\delta(u) \leq \delta(v)$ or equivalently $-\delta(u) + \delta(v) \geq 0$. If $x_u - x_v \geq 2$ it is trivial to derive that

$l\left(\mathbf{y}\right) > l\left(\mathbf{x}\right)$.
